# Association between appendicular skeletal muscle mass as assessed by bioelectrical impedance analysis and airflow limitation in patients with COPD: a cross-sectional study

**DOI:** 10.36416/1806-3756/e20260125

**Published:** 2026-08-13

**Authors:** Jhonata de Marco Vieira, Rosemeri Maurici da Silva

**Affiliations:** 1. Departamento de Medicinal, Universidade Federal de Santa Catarina, Florianópolis (SC) Brasil.

## TO THE EDITOR:

Skeletal muscle mass loss is frequently observed in individuals with COPD and is associated with poorer functionality, reduced quality of life, increased risk of hospitalizations, and higher mortality.^1^ ^,^ ^2^ Factors such as chronic inflammation, physical inactivity, hypoxemia, and comorbidities contribute to muscle mass loss in this population.^3^ ^-^ ^5^ Although low body weight or reduced BMI is often associated with muscle loss, evidence indicates that patients with normal weight, or even those who are overweight/obese, may also present with muscle mass depletion.^2^ ^,^ ^6^ ^,^ ^7^ Therefore, BMI alone may be insufficient to detect early alterations in body composition, given that it does not distinguish between fat and lean mass, potentially overlooking early stages of muscle impairment preceding sarcopenia. 

To investigate whether airflow limitation is associated with low appendicular skeletal muscle mass (ASMM), as well as to explore the level of agreement between ASMM and BMI, we conducted a cross-sectional study of 106 COPD patients followed at a pulmonology outpatient clinic. ASMM was estimated by bioelectrical impedance analysis, alongside assessment of clinical and anthropometric variables. ASMM values below 20 kg for men and below 15 kg for women were considered indicative of low muscle mass, in accordance with criteria described by the European Working Group on Sarcopenia in Older People.^2^ Post-bronchodilator FEV_1_ was categorized as < 50% or ≥ 50% of the predicted value on the basis of the GOLD classification to represent severe airflow limitation. This categorization was adopted to facilitate clinical interpretation of disease severity. Its association with low ASMM was analyzed by means of binary logistic regression adjusted for age, selected a priori on the basis of its clinical relevance as a potential confounder of muscle mass. 

**Figure 1:**
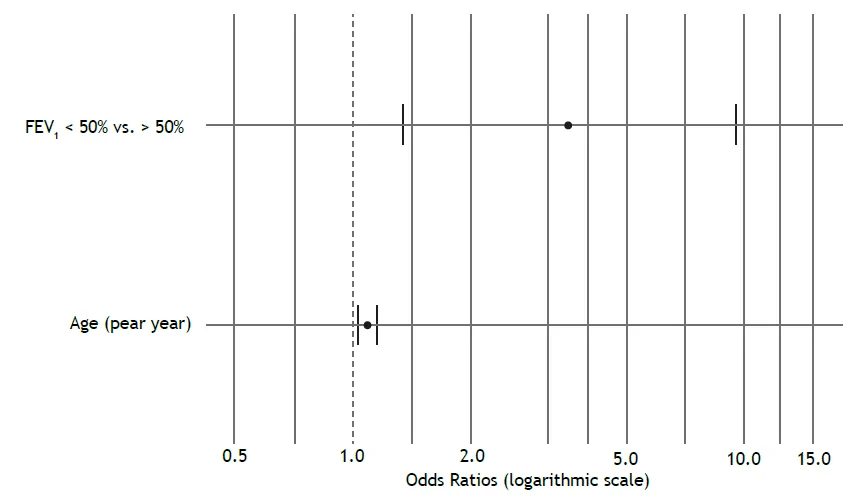
Forest plot showing odds ratios and 95% confidence intervals for factors associated with low appendicular skeletal muscle mass in patients with COPD.

**Figure 2:**
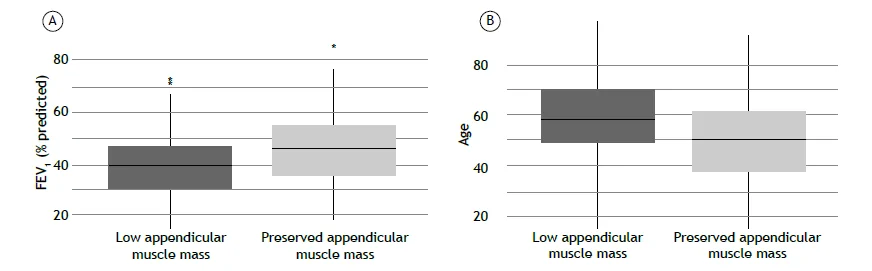
A. Boxplot showing the distribution of post-bronchodilator FEV_1_ by appendicular skeletal muscle mass status (low vs. preserved). Data are presented as median [IQR]. B. Boxplot showing the distribution of age by appendicular skeletal muscle mass status (low vs. preserved). Data are presented as median [IQR].

The sample was predominantly male (56.6%), with a mean age of 66.8 ± 8.3 years. The prevalence of low ASMM was 36.8%, being consistent with previous studies reporting prevalences between 20% and 40% in outpatient COPD populations.^1^ ^,^ ^3^ ^,^ ^5^ Individuals with an FEV_1_ < 50% of the predicted value were more likely to have low muscle mass (OR = 3.58; 95% CI, 1.34-9.57; p = 0.011), independently of age (**Figure 1**). Age was also associated with low muscle mass (OR = 1.09; 95% CI, 1.03-1.15; p = 0.003), indicating a 9% increase in risk per additional year. The distribution of post-bronchodilator FEV_1_ and age by ASMM status is shown in Figure 2. 

Although an association between BMI and low muscle mass was observed (Pearson’s chi-square = 28.010; p < 0.001), the agreement between BMI and ASMM was weak and not statistically significant (kappa = 0.092; p = 0.064), indicating poor concordance between these measures. Notably, 43.6% of patients with low muscle mass were normal weight, showing that BMI alone does not adequately identify muscle mass depletion. Reduced FEV_1_ was associated with low muscle mass, suggesting that greater airflow limitation may reflect systemic manifestations of COPD. However, causality cannot be inferred, because of the cross-sectional design of the study.^8^

Our findings may represent an early stage in the spectrum of muscle dysfunction in patients with COPD, in whom reductions in ASMM precede overt sarcopenia and are associated with functional impairment, supporting a vicious cycle of muscle loss, symptom worsening, and reduced physical activity. In this context, ASMM may serve as a sensitive marker of early systemic involvement in patients with COPD, highlighting the importance of body composition assessment in clinical practice, given that BMI does not differentiate between fat and lean mass and may overlook early muscle impairment. An important limitation of the present study is that although muscle strength was assessed by handgrip dynamometry, values consistent with muscle weakness were not observed. Thus, it was impossible to establish a full diagnosis of sarcopenia in accordance with current European Working Group on Sarcopenia in Older People consensus criteria. Additionally, the wide confidence interval observed in the association between airflow limitation and low muscle mass suggests limited precision of the estimate, despite a sample size of 106 patients. This may be due to sample size constraints or variability in the data and should be considered when interpreting the findings of the present study. Furthermore, the categorization of continuous variables, such as FEV_1_ and BMI, may have resulted in loss of information and reduced statistical power, both of which should also be considered when interpreting the findings of the present study. 

In conclusion, low ASMM was associated with more severe airflow limitation after adjustment for age. Furthermore, BMI showed no significant agreement with low muscle mass, reinforcing its limitations as a screening tool. These findings highlight ASMM as an early marker of muscle impairment, supporting its potential role in the early identification of patients at risk of sarcopenia and related functional decline. 
